# Association between serum uric acid and metabolic syndrome: a cross-sectional study in Bangladeshi adults

**DOI:** 10.1038/s41598-020-64884-7

**Published:** 2020-05-12

**Authors:** Nurshad Ali, Rakib Miah, Mahmudul Hasan, Zitu Barman, Ananya Dutta Mou, Jaasia Momtahena Hafsa, Aporajita Das Trisha, Akibul Hasan, Farjana Islam

**Affiliations:** Department of Biochemistry and Molecular Biology, Shahjalal University of Science and Technology, Sylhet, 3114 Bangladesh

**Keywords:** Metabolic disorders, Metabolic syndrome

## Abstract

Elevated levels of serum uric acid (SUA) have been suggested to associate with cardiovascular disease, diabetes and metabolic syndrome (MetS). However, information is limited on the association between SUA and MetS in general adults. This study aimed to assess the relationship of SUA with MetS and its components in general adults in Bangladesh. A total of 420 participants were enrolled in this study and biochemical parameters including SUA, fasting blood glucose (FBG) and lipid profile were analyzed using standard methods. The NECP criteria were applied to define MetS. The association of SUA with MetS and its components were evaluated by multinomial logistic regression models. The overall prevalence of MetS was 22% with 21.9% in males and 22.1% in female participants. Male subjects had a high prevalence of elevated components of MetS than in the female subjects (p < 0.05 for all cases). The mean concentration of SUA was significantly higher in subjects of the MetS group compared to the non-MetS group (p < 0.05). The components of MetS were raised with the increasing concentrations of SUA across the quartiles. In regression analysis, SUA was significantly associated with the prevalence of MetS in Bangladeshi adults. In conclusion, elevated SUA was significantly associated with the prevalence of MetS and its components.

## Introduction

Metabolic syndrome (MetS) consist of several risk factors including central obesity, elevated blood pressure, hyperglycemia, high triglycerides, and reduced high-density lipoprotein cholesterol^1^. MetS is associated with the increased risk of type 2 diabetes, cardiovascular disease (CVD) and mortality^2,3^. Several population-based studies showed an increased risk of CVD in individuals with MetS compared to those who do not have the syndrome^4,5^. Besides the traditional risk factors, other factors including microalbuminuria, inflammatory markers and hyperuricemia have been suggested to be involved in the MetS^6–8^. Along with MetS, obesity has also been found as an important risk factor for CVD. Furthermore, a link has been found between obesity and hyperuricemia in various studies^9–11^. The prevalence of MetS is increasing at an alarming rate both in developed and developing countries. MetS is highly prevalent among Bangladeshi adults and has been increased rapidly in the last few decades. A recent review reported a high prevalence of MetS (30%) in the Bangladeshi population with 32% in females and 25% in males^12^.

Uric acid in serum is the final oxidation product of purine metabolism in human^13^. Recent epidemiological studies have demonstrated an association of serum uric acid (SUA) with MetS and its components in different populations^14–19^. Some other studies have also found that elevated SUA levels are an independent predictors of the components of MetS, such as high blood pressure and hyperglycemia^20^. However, information is limited regarding the relationship of SUA with MetS in general adults. Moreover, no study has been conducted to examine the association between SUA and MetS in Bangladeshi adults. Given the increased prevalence of MetS in the Bangladeshi population, this cross-sectional study aimed to investigate the relationship of SUA with MetS and its components in general adults. This study also aimed to assess whether SUA is an additional component of MetS in this population.

## Materials and methods

### Study population

This study was a cross-sectional design conducted between November 2017 and May 2019 at the Department of Biochemistry and Molecular Biology of Shahjalal University of Science and Technology, Sylhet, Bangladesh. A total of 420 general adults (aged ≥ 18 years) were enrolled from university students, academic and non-academic staff and local city people of the Sylhet and Dhaka region of Bangladesh. The inclusion criteria were: both gender, aged above 18 years, free from severe chronic illness and willing to participate. Exclusion criteria were: pregnant women, lactating mother and participants with a history of hepatotoxic drug intake, kidney disease, alcohol intake and self-reported evidence of acute or chronic hepatitis. We also excluded participants with missing anthropometric data or blood samples. This study was approved by the Internal Ethics Committee at the Department of Biochemistry and Molecular Biology of Shahjalal University of Science and Technology, Bangladesh. All participants provided written informed consent before inclusion in the study. All steps in the methods section were performed in accordance with the relevant guidelines and regulations.

### General data collection

A standard questionnaire was used to collect demographic and lifestyle information from the participants. Individual anthropometric data such as age, gender, weight and height were recorded in the questionnaire form followed a standard procedure described elsewhere^21–26^. Briefly, systolic and diastolic blood pressure (SBP and DBP) were measured twice in the left arm of the participants with an automated sphygmomanometer (Omron M10, Omron Corporation, Tokyo, Japan) in the seated position after at least 10 minutes of rest. Body mass index (BMI) was calculated as weight in kilograms divided by height in square meters (kg/m^2^). Waist circumference (WC) was measured using general tape that was placed midway between the lowest border of the ribs and iliac crest. Hip circumference (HC) was measured at the largest circumference of the buttocks to the nearest 0.5 cm. Waist-hip ratio (WHR) was measured as waist circumference divided by hip circumference.

### Blood sample collection and laboratory measurements

Venous blood samples were collected after an overnight fast from each subject. The blood samples were centrifuged and stored the isolated serum at −20 °C until laboratory analysis. Serum uric acid (SUA), fasting blood glucose (FBG), total cholesterol (TC), triglycerides (TG), high-density lipoprotein cholesterol (HDL-C), and low-density lipoprotein cholesterol (LDL-C) were measured by colorimetric methods with a semi-auto biochemistry analyzer (Humalyzer 3000, USA) described elsewhere^21,22,26^. The diagnostic kits were purchased from Human Diagnostic (Germany) for analysis of the above clinical parameters. The measurements were carried out according to the standard manufacturer’s protocols provided within the kit. The precision of the measurements was maintained regularly by method standard calibration.

### Diagnostic criteria

In present study, hyperuricemia was defined as SUA concentration >7.0 mg/dL (416.4 µmol/L) in men or >6.0 mg/dL (356.9 µmol/L) in women^27,28^. All volunteers were stratified into four quartiles based on SUA concentrations (Q1: ≤ 243.9, 244–309.3, 309.4–380.7 and> 380.7 µmol/L). Metabolic syndrome (MetS) was diagnosed according to the National Cholesterol Education Program – Adult Treatment Panel III (NCEP-ATP III) criteria^29^. The components of the MetS were defined as following: i) Elevated BP (SBP ≥ 130 mmHg and/or DBP ≥ 85 mmHg or intake of an antihypertensive medication); ii) raised WC (> 102 cm for males and> 88 cm for females); iii) hyperglycemia (FBG ≥ 100 mg/dL). iv) hypertriglyceridemia (TG ≥ 150 mg/dL); v) low HDL-C (< 40 mg/dL for males and <50 mg/dL for females) and subjects with at least three of the above components were identified as having MetS.

### Statistical analysis

Statistical data analyses were performed using IBM SPSS version 23. Data are presented as mean ± SD and quartile ranges. The tests applied during data analysis already described in our previous studies^21,26^. In brief, the baseline characteristics of the volunteers in the SUA quartiles were compared by one-way ANOVA. A Chi-square test was applied to differentiate the proportions of the categorical variables. Differences in the anthropometric and baseline characteristics between the gender groups were done by an independent sample t-test. Pearson’s correlation coefficient test was performed to assess the relationships between baseline variables and SUA concentrations. The association between MetS and SUA levels was evaluated by multinomial logistic regression models. MetS was categorized as yes (presence) and no (absence). In regression analysis, MetS (yes) was considered as the dependent variable and SUA as the independent variable. SUA and other covariates were used as continuous variables in the regression models. We applied three models in the regression analysis. Model 1 was adjusted for age (years) and gender (male and female). Model 2 was adjusted for age, gender and BMI (kg/m^2^), Model 3 was further adjusted for variables used in model 1 and 2 and LDL-C (mg/dL). A p-value of <0.05 was considered statistically significant.

## Results

### Baseline characteristics of the participants in the MetS and non-MetS group

In total, 420 participants (257 male and 163 female) were enrolled in the present study. The baseline characteristics of the participants in the MetS and non-MetS groups are presented in Table 1. Among the participants, 93 subjects were diagnosed with MetS according to the diagnostic criteria. There were significant differences in the mean of age, WC, HC, WHR, BMI, SBP, DBP, FBG, TG, TC, LDL-C (p < 0.001 for all cases) between the MetS and non-MetS groups. The average level of SUA was also higher in the MetS group compared to the non-MetS group (p < 0.05). In contrast, subjects in the non-MetS group had a higher level of HDL-C level than the subjects in the MetS group (p < 0.05).

**Table 1:** Baseline characteristics of the participants based on the presence of MetS.

	Total	MetS	Non-MetS	p-value
*N*	420	93	327	—
Gender, m/f	420	57/36	200/127	—
Age, year	30.5 ± 12.4	39.5 ± 14.1	27.8 ± 10.4	0.000
WC, cm	83.9 ± 10.6	90.5 ± 12.3	81.4 ± 8.6	0.000
HC, cm	92.8 ± 8.2	96.0 ± 8.6	91.5 ± 7.6	0.000
WHR	0.68 ± 0.39	0.91 ± 0.19	0.62 ± 0.41	0.000
BMI, kg/m^2^	23.9 ± 3.8	26.3 ± 3.8	23.3 ± 3.6	0.000
SBP, mmHg	118.2 ± 15.1	128.8 ± 18.1	115.1 ± 12.5	0.000
DBP, mmHg	73.6 ± 14.9	81.6 ± 8.4	71.4 ± 15.6	0.000
SUA, µmol/L	319.2 ± 107.8	332.0 ± 111.8	308.2 ± 106.6	0.026
FBG, mg/dL	98.8 ± 37.5	130.4 ± 66.2	89.9 ± 17.7	0.000
TG, mg/dL	135.1 ± 101.6	211.6 ± 107.2	113.5 ± 88.9	0.000
TC, mg/dL	157.7 ± 63.9	189.2 ± 75.7	148.8 ± 56.3	0.000
HDL-C, mg/dL	31.9 ± 17.1	29.6 ± 11.1	33.4 ± 18.5	0.046
LDL-C, mg/dL	93.3 ± 58.3	115.9 ± 73.2	85.9 ± 50.4	0.000

MetS: Metabolic Syndrome. Values are presented as mean ± SD. P-values are obtained from independent sample t-test.

### Prevalence of MetS and its components in gender and hyperuricemic group

**Figure 1:**
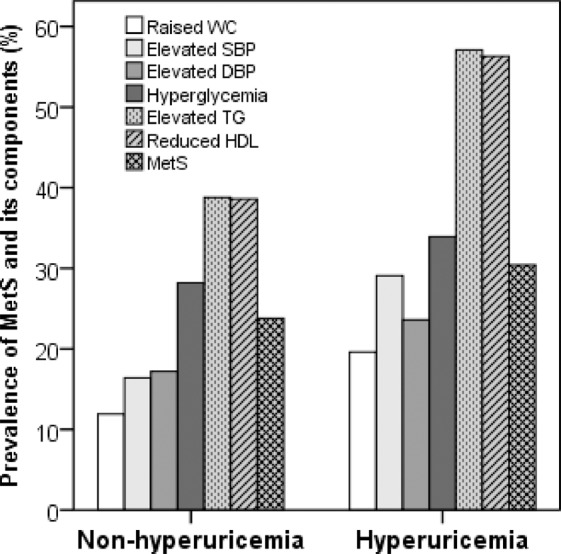
Prevalence of MetS and its components in the hyperuricemic and non-hyperuricemic group. P < 0.05 for all cases when the prevalence of MetS and its component are compared between the groups. P-values are obtained from the Chi-square test.

The prevalence of MetS and its components are presented in Table 2 and Fig. 1. Overall, the prevalence of MetS was 22% with 21.9% in males and 22.1% in female subjects. The MetS components such as raised WC, high blood pressure, hyperglycemia and high TG were significantly higher in male than in the female subjects (p < 0.01 for all cases). In contrast, there was no significant difference for low HDL-C between the male-female groups. The prevalence of hyperuricemia was 16.6% with significant differences between the male (21.3%) and female (8.3%) group (p < 0.01). All components of MetS were significantly higher in subjects in the hyperuricemic group compared to the subjects in the non-hyperuricemic group (p < 0.05 for all cases).

**Table 2:** Prevalence of MetS and its components in the male-female group.

Parameters	Total	Male	Female	P-value
MetS, %	22.0	21.9	22.1	>0.05
Raised WC, %	13.3	4.6	30.9	<0.01
Elevated SBP, %	17.9	24.1	8.6	<0.01
Elevated DBP, %	17.4	18.4	15.9	<0.05
Hyperglycemia, %	25.7	23.4	29.2	<0.05
Elevated TG, %	40.4	52.2	22.9	<0.01
Reduced HDL-C, %	80.7	79.5	82.5	>0.05
Hyperuricemia	16.6%	21.3	8.3	<0.01

The difference between male and female is expressed as p-value. P-values are obtained from Chi-square test.

### Baseline characteristics of the study subjects according to SUA quartiles

The baseline characteristics of the study participants were also evaluated according to SUA quartiles (Table 3). The volunteers were divided into 4 groups based on SUA levels (Q1: ≤ 243.9; Q2: 244–309.3; Q3: 309.4–380.7 and >380.7 µmol/L). No significant difference was observed in the mean age across the SUA quartiles. Participants in the fourth quartile of SUA had a significantly higher WC, WHR, BMI, SBP, DBP, SUA, TG, TC LDL-C and lower FBG and HDL-C than the subjects in the other quartiles of SUA (p < 0.05 for all cases).

**Table 3:** Baseline characteristics of the participants according to SUA quartiles.

	Q1 ≤ 243.9 µmol/L	Q2 244–309.3 µmol/L	Q3 309.4–380.7 µmol/L	Q4> 380.7 µmol/L	p-value for trend
*N*	104	110	105	101	—
Sex, m/f	34/70	57/53	80/25	86/15	—
Age, year	34.1 ± 13.9	31.2 ± 11.9	31.2 ± 12.0	31.3 ± 12.3	0.338
WC, cm	81.9 ± 9.9	82.6 ± 9.7	83.8 ± 8.0	87.3 ± 13.5	0.010
HC, cm	91.7 ± 8.4	92.0 ± 8.5	93.2 ± 6.8	94.1 ± 8.7	0.282
WHR	0.72 ± 0.36	0.75 ± 0.34	0.78 ± 0.31	0.88 ± 0.22	0.010
BMI, kg/m^2^	23.9 ± 3.7	24.2 ± 3.8	24.6 ± 3.5	25.4 ± 3.9	0.008
SBP, mmHg	114.7 ± 15.1	116. 7 ± 13.9	120.5 ± 13.0	124.9 ± 15.2	0.000
DBP, mmHg	69.2 ± 21.1	73.7 ± 12.0	76.0 ± 12.1	77.3 ± 11.9	0.003
SUA, µmol/L	196.2 ± 38.7	282.0 ± 19.7	347.1 ± 20.9	465.6 ± 85.1	0.000
FBG, mg/dL	108.7 ± 55.6	102.7 ± 44.4	90.7 ± 15.8	98.2 ± 28.6	0.025
TG, mg/dL	122.2 ± 73.9	145.9 ± 96.5	156.1 ± 92.4	171.6 ± 122.7	0.012
TC, mg/dL	139.4 ± 71.1	147.4 ± 47.3	164.1 ± 54.9	188.7 ± 77.6	0.000
HDL-C, mg/dL	35.9 ± 12.3	37.7 ± 14.6	31.9 ± 12.1	27.1 ± 13.9	0.000
LDL-C, mg/dL	80.6 ± 68.4	80.9 ± 42.1	98.9 ± 52.7	119.6 ± 66.6	0.000

Values are presented as mean ± SD. P-values are obtained from one-way ANOVA.

### Association of SUA with the prevalence of MetS and its components

Taking SUA as the independent variable and MetS as the dependent variable, multinomial logistic regression was performed to assess the relationship between SUA and MetS. The detailed results are presented in Table 4. In regression analysis, a positive association was observed between SUA and MetS. In all regression models, SUA showed a significant association with the prevalence of MetS. We further assessed the relationship of SUA with the individual components of MetS (Table 5). After adjustment for age, a positive association was observed between SUA and the components of MetS except for hyperglycemia and low HDL-C.

**Table 4:** Multinomial logistic regression analysis to evaluate the association between SUA levels and MetS.

	*B*	SE	Wald	df	OR (95% CI)	P-value
Model 1	0.011	0.003	10.570	1	1.011 (1.004–1.017)	0.001
Model 2	0.008	0.004	5.727	1	1.008 (1.002–1.016)	0.016
Model 3	0.007	0.004	3.872	1	1.006 (1.000–1.013)	0.042

Dependent variable is MetS (yes) and independent variable is SUA (µmol/L). Reference category is normal (non-MetS). Model 1: adjusted for age (years) and gender (male and female). Model 2: model 1+ BMI (kg/m^2^) Model 3: model 2+ LDL (mg/dL). OR, odds ratio; CI, confidence interval; SE, Standard error.

**Table 5:** Age-adjusted logistic regression analysis to evaluate the association between SUA and the components of MetS.

	*B*	SE	Wald	df	OR (95% CI)	P-value
Abdominal obesity	0.009	0.004	6.008	1	1.009 (1.002–1.017)	0.014
High blood pressure	0.008	0.004	4.445	1	1.008 (1.001–1.016)	0.035
Hyperglycemia	−0.001	0.001	0.638	1	0.999 (0.997–1.001)	0.425
High TG	0.11	0.003	17.956	1	1.011 (1.006–1.016)	0.000
Low-HDL-C	0.002	0.002	1.024	1	1.002 (0.998–1.007)	0.312

The dependent variable is MetS components (yes) and the independent variable is SUA (µmol/L). The reference category is normal. The model is adjusted for age (years). OR, odds ratio; CI, confidence interval; SE, Standard error.

## Discussion

The present study investigates the relationship between SUA and MetS in general adults. Although, the association of SUA with MetS has been studied in diabetic and hypertensive subjects, however, limited studies have documented the information regarding the link of SUA with MetS in general adults. In this study, we first report a positive association of SUA with MetS and its components in general adults in Bangladesh.

In the present study, no significant difference was observed in the prevalence of MetS between the gender groups. Among the MetS components, high TG and reduced-HDL-C were the more common abnormalities found in the participants. Anthropometric variables, lipid profile, FBG and SUA were significantly higher in subjects with MetS than in the subjects who do not have this syndrome. In this study, we observed an increasing trend in the levels of individual components of MetS across the SUA quartiles. These results are consistent with the findings reported in Japanese^30,31^, Chinese^19^, Iranian^17^, the United States^32,33^, and European population^34^.

Several epidemiological studies demonstrated a positive association between SUA and the prevalence of MetS in human cohorts. However, it is still debated whether increased SUA concentration is a risk factor or only a biomarker in the progression and development of MetS^19^. Some studies reported that hyperuricemia may be an individual component of the MetS^35,36^, whereas, other works suggested to include hyperuricemia as an additional component of MetS^37,38^. In prospective studies, SUA at baseline level was associated with an increased risk of MetS in both genders^27,39^. A previous study reported that subjects with hyperuricemia have a higher chance of developing of MetS than in the non-hyperuricemic subjects^40^. This predictive role of SUA has been demonstrated in individuals who were free of all components of MetS at baseline^27^. Moreover, a recent clinical study reported that elevated levels of SUA can play a pathogenic role in MetS^41^. Due to controversial findings, NCEP could not include hyperuricemia as an individual component of MetS yet, and it seems that further longitudinal investigations are required to elucidate whether hyperuricemia is another component of the MetS or not^17^.

In regression models, SUA showed a significant association with MetS and several components of MetS. However, after adjusting some confounding factors, we did not find the significant association of SUA with hyperglycemia and low HDL-C. This might be happened because of the inverse relationship of SUA with the prevalence of diabetes in the Bangladeshi population^26^. On the other hand, we observed a very high prevalence of low HDL-C among the participants that could be a reason for the non-significant association of SUA with HDL-C.

The underline mechanisms between SUA and MetS are not fully understood yet. The possible mechanisms of SUA in inducing MetS are as follows: First, hyperuricemia has been demonstrated to cause endothelial dysfunction in human and animal models^42,43^. Second, SUA has been exhibited to hinder the production of NO^44^, which is considered as important for insulin function^45,46^. Deficiency of endothelial-formed NO is thought to reduce blood flow to cells leading to stop the insulin action and inducing hyperinsulinemia^17^. Thus, hyperuricemia may have a potential role in inducing or worsening insulin resistance by itself. In turn, the resistance of insulin is believed to play a vital role in the pathogenesis of MetS^47^. Although a positive association of hyperuricemia with MetS has been demonstrated through epidemiological and animal studies, the exact mechanisms by which SUA leads to this disorder are still at the beginning need to be explained. Thus, it is obvious that further prospective studies are required to determine the role of SUA in the development of MetS.

There were some limitations to the study. First, the sample size in the present study was relatively small. Second, the cross-sectional nature of the data did not allow us to draw a causal relationship between SUA and MetS. Moreover, we adjusted some probable confounding factors in determining the relationship between SUA and MetS; however, other confounding factors effects cannot be excluded. For example, we did not have information on renal function tests and diet habits that can affect SUA levels. Therefore, the present study findings should be generalized cautiously to other general populations.

## Conclusions

The present study showed that the components of MetS are raised with the increasing concentrations of SUA across the quartiles. In this study, SUA was significantly associated with the prevalence of MetS and its components. Given the high prevalence of MetS among Bangladeshi adults, more studies are required to examine the role of SUA in the pathogenesis of MetS. Furthermore, attention should be paid to the increased risk of MetS and hyperuricemia for the general population.

## Data Availability

All related data are included in this paper. The datasets produced and/or analyzed during the present study are available from the corresponding author upon reasonable request.
